# SUICIDE RISK ASSESSMENT: INSIGHTS, CHALLENGES, AND INNOVATIONS

**DOI:** 10.1093/geroni/igae098.1834

**Published:** 2024-12-31

**Authors:** Fatema Colombowala, Kimberly Van Orden

**Affiliations:** Chair; Discussant

## Abstract

Suicide is a major public health problem, accounting for over 700,000 deaths globally each year. Worldwide, suicide rates are highest among adults 70 years and older. Suicide attempts in later life are highly lethal and many who die by suicide do not endorse suicidal ideation, challenging efforts to detect and manage risk. This symposium will provide an in-depth exploration of suicide risk assessment amongst older adults, highlighting unique challenges across settings and providing recommendations for best practices and future research. Speaker 1 will present on the acceptability and feasibility of using ecological momentary assessment (EMA) to measure daily fluctuations in suicidal thoughts and psychosocial risk factors amongst older adults with subjective cognitive decline. Speaker 2 will discuss challenges of assessing suicidality within rural in-home care settings amongst older adults with hoarding disorder, emphasizing safety considerations when conducting in-home suicide risk assessments. Speaker 3 will examine the relationship between hoarding disorder and suicidal ideation, emphasizing the impact of social cohesion risk factors in rural environments. Speaker 4 will share findings from a comparative analysis of two validated suicide assessment tools (clinician-administered versus self-report) commonly used in research and practice, with a focus on areas of differential response across the two scales. The Discussant will synthesize findings and highlight areas for future research and innovation to improve detection of suicidal ideation and behavior amongst older adults. Collectively, these presentations will offer insights into the complexities of suicide risk assessment and recommend strategies tailored to the needs of older adults.

